# Effects of Sample Matrix, RNA Extraction Method, Carrier RNA, and Storage Conditions on RT-qPCR Detection of Foot-and-Mouth Disease Virus

**DOI:** 10.3390/vetsci13101096

**Published:** 2026-10-15

**Authors:** Vesna Milićević, Bojan Milovanović, Zorana Zurovac Sapundžić, Sofija Šolaja, Dimitrije Glišić, Đorđe Aksić, Ljubiša Veljović, George Valiakos

**Affiliations:** 1 Institute of Veterinary Medicine of Serbia, Janisa Janulisa 14, 11000 Belgrade, Serbia; bojan.milovanovic@nivs.rs (B.M.); zorana.zurovac@nivs.rs (Z.Z.S.); sofija.solaja@nivs.rs (S.Š.); dimitrije.glisic@nivs.rs (D.G.); djordje.aksic@nivs.rs (Đ.A.); ljubisa.veljovic@nivs.rs (L.V.); 2 Faculty of Veterinary Medicine, University of Thessaly, Trikalon 224, 43131 Karditsa, Greece; georgevaliakos@uth.gr

**Keywords:** foot-and-mouth disease virus, RNA extraction, RT-qPCR, carrier RNA, RNA stability, sample matrix

## Abstract

Reliable molecular detection of foot-and-mouth disease virus (FMDV) depends on RT-qPCR performance and pre-analytical factors, including sample matrix, RNA extraction method, carrier RNA supplementation, and storage conditions. This study compared silica membrane- and magnetic bead-based RNA extraction workflows for detection of FMDV serotypes O, A, and SAT2 in serum, PBS-based swab suspension, and tissue homogenates at three virus dilutions, with and without carrier RNA. FMDV RNA stability in serum stored at room temperature or −20 °C for up to 60 days and following five freeze–thaw cycles was also evaluated using serotype O. Extraction performance was matrix-dependent. In serum, silica-based workflow yielded significantly lower Ct values than magnetic bead-based extraction. In tissue homogenates, magnetic bead extraction yielded significantly lower Ct values with carrier RNA, whereas carrier RNA impaired silica-based extraction. No significant differences were observed for PBS-based swab suspension samples. FMDV RNA detectability remained stable at −20 °C for 60 days, whereas room-temperature storage caused progressive signal loss, which was reduced by carrier RNA. Five freeze–thaw cycles did not progressively affect RNA detectability. These findings demonstrate that the effects of extraction workflow and carrier RNA supplementation on FMDV RT-qPCR detectability differed according to the sample matrix and experimental conditions, highlighting the importance of matrix-specific optimization of pre-analytical procedures.

## Simple Summary

Foot-and-mouth disease is a highly contagious viral disease of livestock, and rapid and reliable laboratory detection is essential for disease control. However, the accuracy of molecular testing can be affected by the type of sample, the method used to extract viral genetic material, and the conditions under which samples are stored. In this study, we compared two commonly used extraction approaches in serum, swab suspensions, and tissue samples and examined whether adding carrier ribonucleic acid improved virus detection. We also investigated how storage temperature and repeated freezing and thawing affected the ability to detect the virus. The results showed that extraction performance depended on the type of sample, and no single method performed best under all conditions. Carrier ribonucleic acid was beneficial in some combinations but had little effect in others. Viral genetic material remained readily detectable in frozen serum for 60 days, while prolonged storage at room temperature progressively reduced detection. Repeated freezing and thawing up to five times had little effect. These findings can help diagnostic laboratories optimize sample processing and storage procedures and improve the reliability of foot-and-mouth disease diagnosis.

## 1. Introduction

Foot-and-mouth disease (FMD) is a highly contagious transboundary disease affecting domestic and wild cloven-hoofed animals [1]. It is caused by foot-and-mouth disease virus (FMDV), a small, non-enveloped, positive-sense, single-stranded RNA virus belonging to the genus *Aphthovirus* within the family *Picornaviridae* [2]. The virus is classified into seven immunologically distinct serotypes, O, A, C, Asia 1, SAT1, SAT2, and SAT3, between which there is little or no cross-protection [3]. Foot-and-mouth disease remains endemic in large parts of Africa, the Middle East, and Asia, as well as in limited areas of South America, whereas Europe, North and Central America, Australia, New Zealand, and several other regions are free from the disease [4]. Nevertheless, FMD-free countries remain vulnerable to incursions through the movement of infected animals, contaminated animal products, fomites, and other transmission pathways. This continuing threat was demonstrated by the re-emergence of FMD in Germany, Hungary, and Slovakia in 2025, following decades of freedom from the disease in these countries [5]. These outbreaks highlighted the importance of maintaining preparedness and effective diagnostic capacity even in historically FMD-free regions.

Reliable laboratory diagnosis depends not only on the analytical performance of the detection assay but also on the quality, type, and quantity of the submitted sample, as well as its collection, transport, storage, and processing. Field conditions may compromise sample integrity through delayed transport, exposure to unsuitable temperatures, or repeated freezing and thawing. Moreover, samples may need to be stored for extended periods before testing or subsequently used for retrospective analyses. As FMDV has an RNA genome, degradation during sample handling and storage may reduce the amount of detectable target RNA and consequently affect diagnostic sensitivity. Real-time reverse transcription polymerase chain reaction (RT-qPCR) is widely used for rapid FMDV detection because of its high sensitivity, specificity, and suitability for high-throughput testing [6,7].

Real-time RT-PCR has become an important tool for rapid FMDV detection in clinical specimens, although diagnostic performance may be influenced by both assay-related and pre-analytical factors [8]. FMDV-specific studies have demonstrated that RNA extraction and sample matrix can substantially affect molecular detection. In particular, Goris et al., 2009 [9] showed matrix-dependent differences in analytical sensitivity across several experimentally spiked biological matrices, emphasizing the importance of considering extraction conditions and matrix effects during assay validation. Sample preservation may also influence the diagnostic workflow; nevertheless, FMDV RNA has been shown to remain detectable by RT-qPCR even in severely deteriorated vesicular epithelium under certain conditions [10].

However, efficient and reproducible RNA extraction is a prerequisite for reliable molecular detection. Extraction efficiency may be influenced by viral RNA concentration, sample composition, the presence of inhibitors, and the physicochemical principles of the extraction method. Silica membrane- and magnetic bead-based procedures are among the most frequently used nucleic acid extraction approaches in diagnostic laboratories. Although both rely on the selective binding of nucleic acids under appropriate chemical conditions, they differ in their workflows, automation options, throughput, and susceptibility to matrix-related effects. Carrier RNA is frequently used to improve the recovery of small amounts of nucleic acid by enhancing binding and reducing target loss during extraction [11]. However, its effect may vary between extraction systems because silica membrane- and magnetic bead-based methods differ in their binding surfaces, extraction chemistry, washing and elution procedures, and interactions with sample matrix components. These differences may influence the effect of carrier RNA on subsequent RT-qPCR detectability. Consequently, the benefit of carrier RNA may depend on both the extraction workflow and the biological matrix.

Despite widespread use of silica- and magnetic bead-based extraction in FMD diagnostic laboratories, there is limited comparative evidence regarding how their performance varies across clinically relevant matrices and in the presence of carrier RNA supplementation. Therefore, this study aimed to compare the performance of silica membrane- and magnetic bead-based RNA extraction workflows for RT-qPCR detection of FMDV serotypes O, A, and SAT2 in serum, PBS-based swab suspension, and tissue homogenates, and to evaluate the effect of carrier RNA supplementation. In addition, FMDV RNA stability during prolonged storage at room temperature and −20 °C and following repeated freeze–thaw cycles were assessed in serum.

## 2. Materials and Methods

Viruses and sample matrices

Inactivated FMDV serotypes O (O1 Manisa), A (A22 IRQ 24/64), and SAT2 (SAT2 Eritrea), obtained from The Pirbright Institute, United Kingdom, were used to prepare experimentally spiked samples in three matrices: bovine spleen homogenate, bovine serum, and PBS-based swab suspension. The viral preparations were supplied as inactivated reference material, and virus inactivation was not performed as part of the present study. The starting viral RNA concentrations of the three FMDV preparations were not standardized to an equivalent RNA concentration. Therefore, absolute Ct values were not used to compare viral RNA concentrations among serotypes. Instead, each serotype–dilution combination was treated as a matched experimental unit for comparisons between extraction conditions. All biological matrices had previously tested negative for FMDV.

For preparation of the spleen matrix, approximately 3 g of previously stored FMDV-negative bovine spleen tissue was homogenized in 27 mL of phosphate-buffered saline (PBS) at a ratio of 1:10 (*w*/*v*) using pestle and mortar. The homogenates were clarified by centrifugation at 4000 rpm for 10 min, and the resulting supernatants were used as the matrix for virus spiking. Aliquots of 22.5 mL of spleen homogenate were mixed with 2.5 mL of each FMDV preparation to obtain a virus-to-matrix ratio of 1:10 (*v*/*v*).

For preparation of the PBS-based swab suspension matrix, 50 swabs were individually immersed in 1 mL of PBS and vortexed for 2 min. Aliquots of 22.5 mL of the resulting PBS-based swab suspensions were subsequently spiked with 2.5 mL of each virus preparation at a virus-to-matrix ratio of 1:10 (*v*/*v*). Thus, the experimental setup was designed to assess FMDV RNA recovery from a PBS-based swab suspension matrix rather than to reproduce the complete field swab collection procedure.

For the serum matrix, FMDV-negative bovine serum samples were used without additional pretreatment. Aliquots of 40.5 mL of serum were directly spiked with 4.5 mL of each FMDV preparation at a virus-to-matrix ratio of 1:10 (*v*/*v*).

Following preparation of the three spiked matrices, serial 10-fold dilutions from 10^−1^ to 10^−3^, were prepared, and the samples were subsequently subjected to nucleic acid extraction and RT-qPCR analysis as described below.

RNA extraction methods and carrier RNA supplementation

RNA extraction was performed with and without carrier RNA using two extraction methods: a magnetic bead-based method employing the IndiMag Pathogen Kit (INDICAL BIOSCIENCE GmbH, Leipzig Germany), and a silica membrane-based method employing the IndiSpin Pathogen Kit (INDICAL BIOSCIENCE GmbH, Leipzig, Germany). In this experiment, carrier RNA (INDICAL BIOSCIENCE GmbH, Leipzig, Germany) was added as part of the extraction procedure to evaluate its effect on FMDV RNA extraction performance and subsequent RT-qPCR detectability. All extractions were performed according to the manufacturers’ instructions. Magnetic bead-based extraction was automated using the IndiMag 48 instrument (INDICAL BIOSCIENCE GmbH, Leipzig, Germany), whereas silica membrane-based extraction was performed using the QIAcube Connect instrument (QIAGEN, Hilden, Germany). For both extraction methods, 200 µL of sample was used as the starting volume, with 100 µL of lysis buffer and 1 µL carrier RNA (1 µg/µL) per sample in the carrier RNA-supplemented conditions. The final RNA elution volume was 100 µL for both extraction methods. All experimental conditions were tested in triplicate (Supplementary Table S1).

Stability of FMDV RNA under different storage conditions

The stability of FMDV RNA under different storage conditions was evaluated using bovine serum spiked with FMDV O/Manisa at a ratio of 1:10. The spiked serum was divided into two groups: samples supplemented with carrier RNA at a ratio of 1:100 (1 µL carrier RNA, 1 µg/µL, per 100 µL of serum), and samples without carrier RNA. In contrast to the extraction experiment, carrier RNA was intentionally added directly to the serum before storage and was not added during subsequent RNA extraction. This design was used to evaluate whether the presence of carrier RNA during storage influenced the preservation and subsequent RT-qPCR detectability of FMDV RNA, independently of its conventional use during the extraction procedure. Aliquots from both groups were stored either at room temperature (22 ± 3 °C) or at −20 ± 2 °C and analysed on days 0, 3, 5, 30, and 60. At each time point, RNA was extracted using the IndiSpin Pathogen Kit (INDICAL BIOSCIENCE GmbH, Leipzig, Germany) and the QIAcube Connect instrument (QIAGEN, Hilden, Germany). No additional carrier RNA was added during extraction. All experimental conditions were tested in triplicate (Supplementary Table S1).

Effect of repeated freeze–thaw cycles

The effect of repeated freeze–thaw cycles on FMDV RNA detection was evaluated using bovine serum spiked with FMDV O/Manisa. Before freeze–thaw treatment, the spiked serum was divided into two groups: samples supplemented with carrier RNA at a ratio of 1:100 (1 µL carrier RNA, 1 µg/µL, per 100 µL of serum), and samples without carrier RNA. Carrier RNA was added before the first freeze–thaw cycle to assess whether its presence in the sample influenced FMDV RT-qPCR detectability following repeated freezing and thawing. No carrier RNA was added during subsequent RNA extraction, thereby avoiding any additional effect of carrier RNA on extraction efficiency. Samples were subjected to five consecutive freeze–thaw cycles. For each cycle, the same sample tubes were frozen at −20 ± 2 °C for 1 h and subsequently thawed completely at room temperature (22 ± 3 °C). Following thawing, samples were thoroughly mixed before being returned to −20 °C for the subsequent cycle. Following each cycle, RNA was extracted using the IndiSpin Pathogen Kit (INDICAL BIOSCIENCE GmbH, Leipzig, Germany) and the QIAcube Connect instrument (QIAGEN, Hilden, Germany), without the addition of carrier RNA during extraction. All experimental conditions were tested in triplicate (Supplementary Table S1).

FMDV RT-qPCR

RNA extracted in all three experiments was analysed by RT-qPCR using the Luna^®^ Universal Probe One-Step RT-qPCR Kit (New England Biolabs, Ipswich, MA, USA). The assay targeted the conserved 3D region of the FMDV genome and was performed according to the previously published protocol for the molecular detection of FMDV described by Moniwa et al., 2007 [7]. The total reaction volume was 12.5 µL and comprised 1.88 µL of nuclease-free water, 6.25 µL of Luna Universal One-Step Reaction Mix (2×), 0.63 µL of Luna WarmStart^®^ RT Enzyme Mix (20×), 0.50 µL of each primer (10 µM), 0.25 µL of probe (10 µM), and 2.50 µL of RNA template. The thermal cycling conditions consisted of reverse transcription at 55 °C for 10 min, initial denaturation at 95 °C for 1 min, followed by 45 cycles of denaturation at 95 °C for 10 s and annealing/extension at 60 °C for 30 s. Amplification was performed using a QuantStudio™ 3 Real-Time PCR System (Applied Biosystems, Thermo Fisher Scientific, Foster City, CA, USA). Appropriate positive controls, negative extraction controls, and no-template controls were included in each run. The VetMAX™ Xeno™ Internal Positive Control (IPC) RNA (Thermo Fisher Scientific, Foster City, CA, USA) was included as an internal extraction and amplification control to monitor extraction and amplification performance and to identify potential RT-qPCR inhibition. Cycle threshold (Ct) values obtained under the different experimental conditions were used for comparative analysis.

Statistical analysis

Ct values obtained from three technical replicates were summarized as means ± standard deviation (SD). The inferential analysis was restricted to predefined within-matrix pairwise comparisons, with virus serotype–dilution combinations treated as matched experimental units. The three replicate RT-qPCR measurements represented technical rather than independent biological replicates. Consequently, the statistical inference was restricted to the experimental conditions tested and was not intended to estimate biological variability or broader diagnostic reproducibility. Technical repeatability was additionally assessed using the range between the minimum and maximum Ct values within each triplicate. The effects of the extraction method (magnetic bead-based versus silica membrane-based extraction) and carrier RNA supplementation were evaluated separately for serum, PBS-based swab suspension, and tissue matrices. Comparisons were performed using the paired two-sided Wilcoxon signed-rank test, with virus serotype and dilution combinations treated as matched experimental units. Non-detect reactions were treated as missing values and were not assigned an arbitrary Ct value. Paired Wilcoxon signed-rank analyses included only matched experimental units with measurable Ct values under both conditions being compared. Differences were expressed as ΔCt, calculated as the mean Ct under the first condition minus the mean Ct under the reference condition. To account for multiple testing across matrices and experimental contrasts, raw *p*-values were adjusted using the Holm method. An adjusted *p*-value < 0.05 was considered statistically significant.

The stability and freeze–thaw experiments were analysed descriptively because they were based on technical replicates of the same experimental material.

For each storage condition, time point and carrier RNA treatment, results were expressed as mean Ct ± SD. Changes during storage were reported as ΔCt relative to day 0, whereas changes following repeated freeze–thaw cycles were calculated relative to the first cycle. Statistical analyses were performed using Python 3.13 (Python Software Foundation, Wilmington, DE, USA) with the SciPy package 1.17.0.

## 3. Results

Influence of extraction method and carrier RNA on FMDV RNA detection

The performance of magnetic-bead and silica membrane-based extraction was evaluated in serum, PBS-based swab suspension and tissue matrices spiked with FMDV serotypes O, A and SAT2 at three dilutions. Most technical triplicates showed good repeatability, with a median within-triplicate SD of 0.20 Ct. No evidence of substantial RT-qPCR inhibition was observed based on IPC performance. IPC Ct values ranged from 31 to 34 across the tested conditions. Nine reactions, corresponding to three experimental conditions in which all three technical replicates were negative, yielded no detectable Ct value. All non-detectable reactions were obtained for serotype O at the highest dilution in the PBS-based swab suspension matrix, except when silica membrane-based extraction was performed with carrier RNA. Non-detect reactions were treated as missing values and were not assigned an arbitrary Ct value. No mean Ct was calculated for these conditions. The effect of the extraction method varied according to the sample matrix and carrier RNA supplementation. A summary of the key pairwise comparisons across matrices, extraction methods, and carrier RNA conditions is provided in Table 1.

In serum samples extracted without carrier RNA, silica membrane-based extraction workflow yielded consistently lower Ct values than magnetic bead-based extraction, with a mean difference of −6.63 Ct (median ΔCt: −7.67; Holm-adjusted *p* = 0.047). The silica membrane-based workflow also yielded significantly lower Ct values in serum samples extracted with carrier RNA, producing Ct values that were, on average, 3.15 cycles lower than those obtained using magnetic bead-based extraction (median ΔCt: −3.19; Holm-adjusted *p* = 0.047). For magnetic bead-based extraction of serum samples, the addition of carrier RNA reduced the mean Ct by 3.51 cycles compared with extraction without carrier RNA. However, this difference was not statistically significant after adjustment for multiple comparisons (median ΔCt: −4.25; Holm-adjusted *p* = 0.094). Carrier RNA had virtually no overall effect on silica membrane-based workflow of serum samples (mean ΔCt: −0.03; median ΔCt: −0.05; Holm-adjusted *p* = 1.000).

No significant differences between extraction methods or carrier RNA conditions were detected in the PBS-based swab suspension matrix. Without carrier RNA, silica extraction workflow produced Ct values that were, on average, 1.32 cycles lower than those obtained using magnetic extraction workflow (Holm-adjusted *p* = 1.000). In the presence of carrier RNA, the corresponding mean difference was −0.87 Ct (Holm-adjusted *p* = 1.000). Carrier RNA did not significantly affect magnetic bead-based extraction (mean ΔCt: −0.37; Holm-adjusted *p* = 1.000) or silica membrane-based workflow (mean ΔCt: +0.08; Holm-adjusted *p* = 1.000). Interpretation of the PBS-based swab suspension results was limited by the non-detectable reactions for 10^−3^ diluted serotype O and by the variable triplicate identified for the 10^−2^ serotype O dilution. In tissue samples extracted with carrier RNA, magnetic bead-based extraction yielded significantly lower Ct values than the silica membrane-based workflow. The mean Ct obtained using silica extraction was 3.71 cycles higher than that obtained using magnetic extraction (median ΔCt: 3.44; Holm-adjusted *p* = 0.047). In the absence of carrier RNA, no consistent difference between the two extraction methods was detected (mean ΔCt: −0.89; median ΔCt: −0.06; Holm-adjusted *p* = 1.000). The effect of carrier RNA in tissue samples also depended on the extraction method. With magnetic bead-based extraction, carrier RNA supplementation led to a reduction in the Ct by 2.44 cycles on average; however, the difference was not significant after adjustment for multiple comparisons (median ΔCt: −1.29; Holm-adjusted *p* = 0.191). In contrast, the addition of carrier RNA during the silica membrane-based workflow significantly increased the mean Ct by 2.16 cycles (median ΔCt: 2.23; Holm-adjusted *p* = 0.047) (Supplementary Table S2).

Stability of FMDV RNA during storage

**Figure 1:**
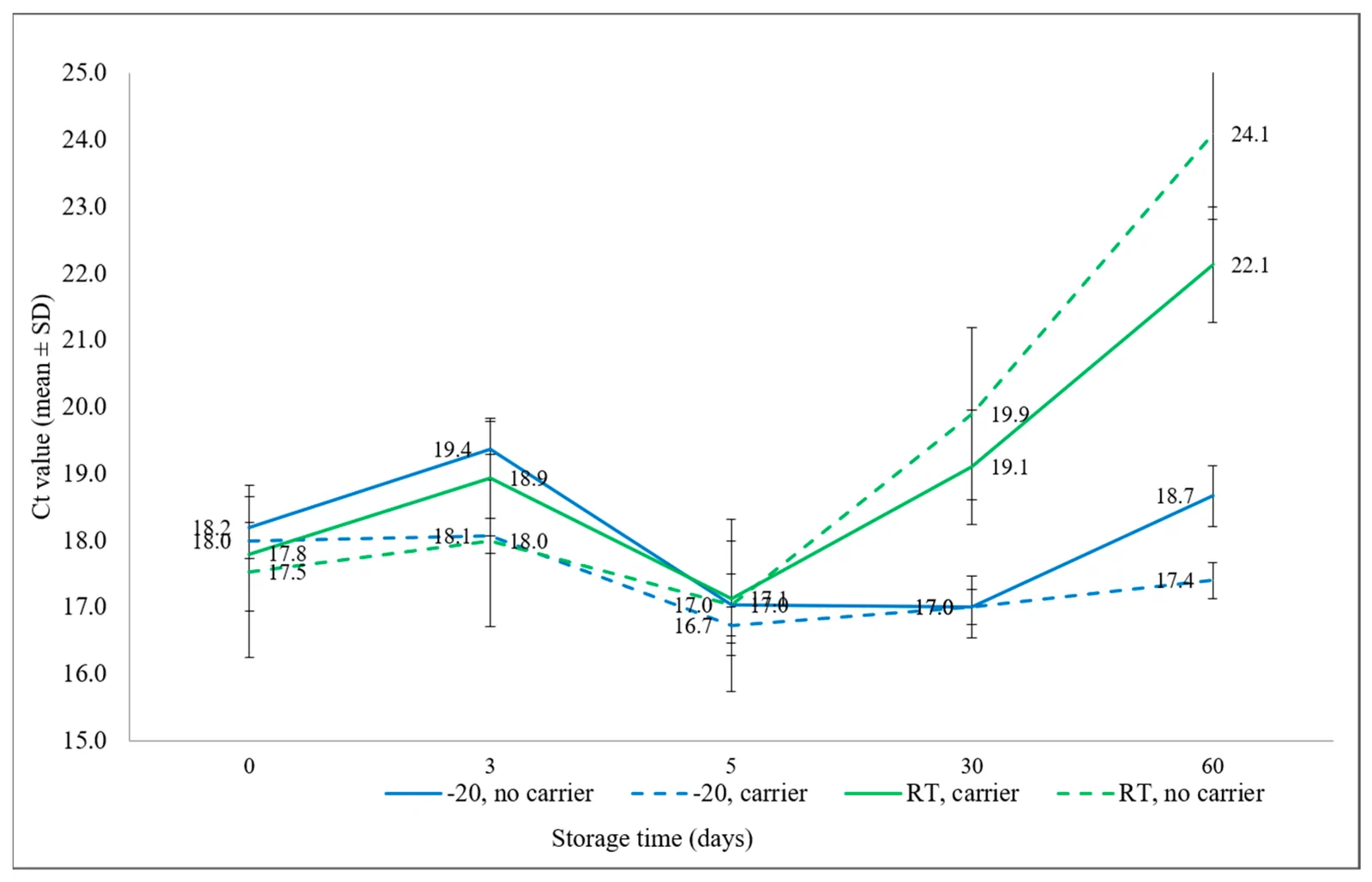
Effect of storage conditions on FMDV RNA detection. Bovine serum was spiked with FMDV serotype O (O1 Manisa) at a 1:10 (10^−1^) virus-to-serum ratio and stored at room temperature (22 ± 3 °C) or −20 ± 2 °C for up to 60 days, with or without carrier RNA supplementation. Mean Ct values were determined on days 0, 3, 5, 30, and 60. Values represent the mean of three technical replicates (n = 3).

Storage temperature had a pronounced effect on the stability of FMDV RNA in serum. At −20 °C, the RT-qPCR signal remained stable throughout the 60-day observation period. After 60 days, the mean Ct changed by only +0.47 cycles in samples supplemented with carrier RNA and by −0.60 cycles in samples without carrier RNA relative to day 0. The fluctuations observed at intermediate time points were not progressive and remained within approximately 1.3 Ct of the baseline values. At room temperature, Ct values increased with prolonged storage, particularly between days 30 and 60. After 30 days, the mean Ct had increased by 1.30 cycles in samples containing carrier RNA and by 2.37 cycles in samples without carrier RNA. After 60 days, the increases reached 4.33 and 6.57 Ct, respectively. After 60 days at room temperature, the increase in Ct was smaller in samples stored with carrier RNA than in those stored without carrier RNA (4.33 versus 6.57 Ct), corresponding to a difference of approximately 2.23 Ct. This finding indicates better preservation of RT-qPCR detectability in the presence of carrier RNA under the tested conditions (Figure 1).

Effect of repeated freeze–thaw cycles

**Figure 2:**
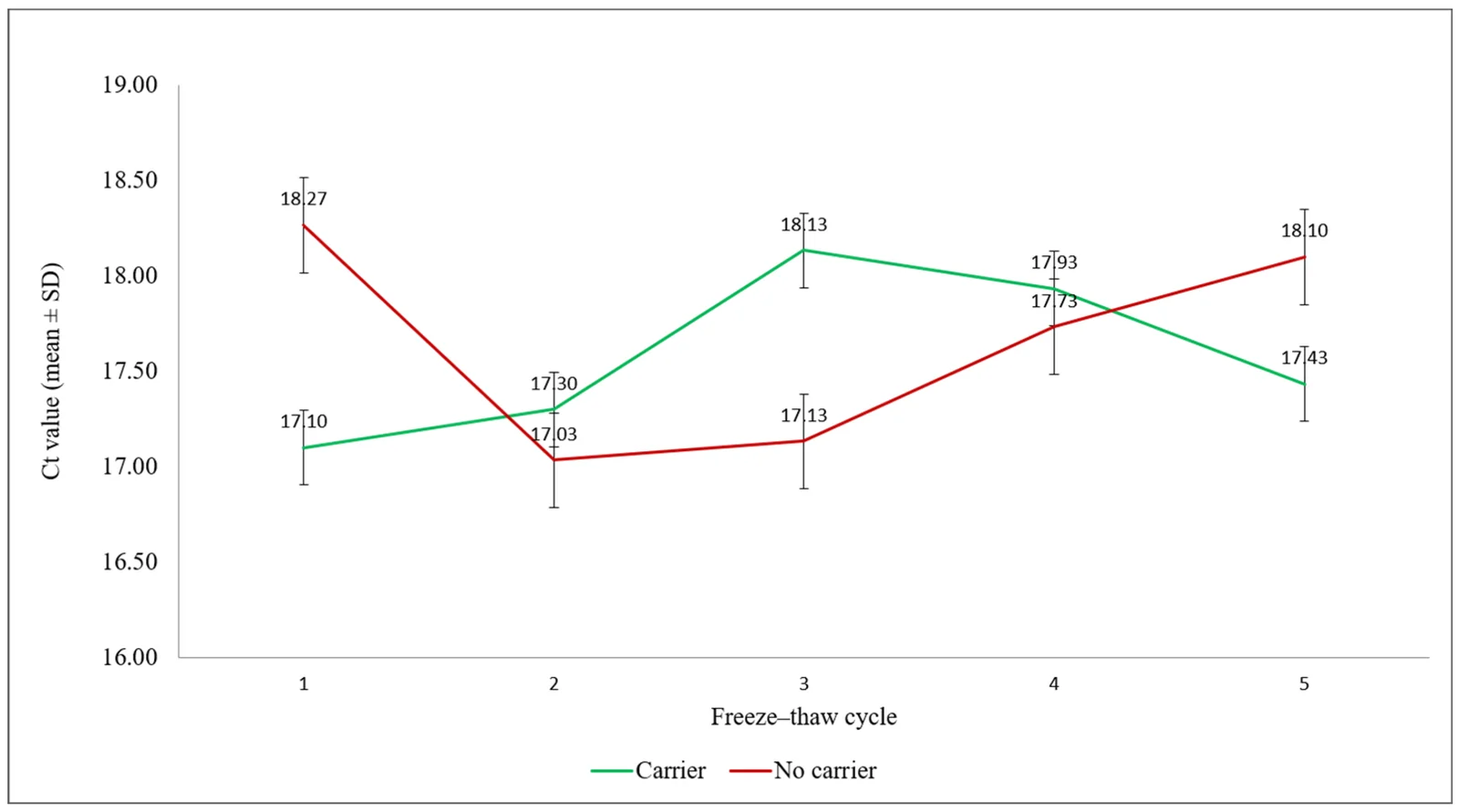
Effect of repeated freeze–thaw cycles on FMDV RNA detection. Bovine serum was spiked with FMDV serotype O (O1 Manisa) at a 1:10 (10^−1^) virus-to-serum ratio and subjected to five consecutive freeze–thaw cycles, with or without carrier RNA supplementation. Each cycle consisted of freezing at −20 ± 2 °C for 1 h followed by complete thawing at room temperature (22 ± 3 °C). Ct values were determined after each cycle. Values represent the mean of three technical replicates (n = 3).

Repeated freezing and thawing did not produce a progressive increase in Ct values over five cycles. In samples containing carrier RNA, the mean Ct increased from 17.10 after the first cycle to 17.43 after the fifth cycle, corresponding to a net change of +0.33 Ct. In samples without carrier RNA, the mean Ct changed from 18.27 after the first cycle to 18.10 after the fifth cycle, representing a net change of −0.17 Ct. Although fluctuations of approximately 1 Ct were observed at some intermediate cycles, there was no evidence of cumulative loss of detectable FMDV RNA under the tested conditions (Figure 2).

## 4. Discussion

The present study demonstrates that the effects of the tested extraction workflows and carrier RNA supplementation on FMDV RT-qPCR detectability differed according to the sample matrix and experimental conditions. Because RNA yield, purity, and extraction efficiency were not independently quantified, differences in Ct values cannot be attributed specifically to differences in RNA recovery.

Three FMDV preparations representing serotypes O, A, and SAT2 were included to assess whether the observed extraction patterns were reproducible across different viral preparations rather than being restricted to a single serotype. However, as only one strain per serotype was evaluated, the study was not designed to assess potential strain- or topotype-specific differences in extraction performance.

The two extraction workflows showed matrix-dependent differences in subsequent FMDV RT-qPCR detectability. Because extraction principle, commercial kit, and automated platform differed simultaneously between the two workflows, the observed differences cannot be attributed specifically to silica membrane-based versus magnetic bead chemistry. No single extraction approach performed optimally across all matrices, emphasizing the need for matrix-specific validation of pre-analytical procedures.

The silica membrane-based extraction workflow consistently produced significantly lower Ct values in serum samples, irrespective of carrier RNA supplementation, whereas magnetic bead-based extraction yielded significantly lower Ct values for tissue homogenates when carrier RNA was included. No consistent difference between extraction methods was observed for PBS-based swab suspension samples. These matrix-dependent differences may partly reflect the physicochemical characteristics of the sample matrices and differences between the nucleic acid capture mechanisms of the two extraction workflows.

The lower Ct values observed with carrier RNA during magnetic extraction of tissue samples may reflect differences occurring during nucleic acid extraction. However, alternative factors, including residual inhibition, matrix effects, or differences affecting subsequent reverse transcription and amplification, cannot be excluded. Thus, the mechanism underlying the increased Ct values observed following carrier RNA supplementation during silica membrane-based workflow of tissue samples remains unclear. Tissue homogenates contain abundant host nucleic acids, proteins, cellular debris, and other matrix components that may interact with the extraction chemistry. The observed increase in Ct values may also reflect matrix-dependent effects on the extraction and downstream RT-qPCR process, including differential carry-over of inhibitory components. As these factors were not directly assessed, the underlying mechanism cannot be determined from the present data. These observations agree with previous studies demonstrating that RT-qPCR detectability depends on the extraction chemistry and sample matrix rather than on the extraction method alone [12,13,14]. In a validation study of two FMDV real-time RT-PCR assays, spiking the same FMDV dilution series into different biological matrices resulted in no detectable matrix effect on analytical sensitivity in blood or foot epithelium, an approximately 1 log10 reduction in detection limit in faecal and tongue epithelium samples, and an approximately 3 log10 reduction in spleen compared with cell-culture controls [9]. More recently, matrix-dependent differences in FMDV RT-qPCR detection have also been reported, with little or no effect on the detection limit in tongue epithelium, saliva, milk, and oro-pharyngeal fluid, but an approximately 1 log10 reduction in EDTA blood [15].

The effect of carrier RNA was likewise dependent on both the extraction workflows and the sample matrix. Although carrier RNA tended to improve RNA RT-qPCR detectability from serum and tissue samples extracted using the magnetic bead-based workflow, these improvements were not statistically significant after correction for multiple comparisons. In contrast, carrier RNA provided no measurable benefit during silica extraction of serum and significantly increased Ct values during silica extraction of tissue samples. These findings indicate that carrier RNA should not be regarded as universally beneficial but rather as a parameter requiring validation for each extraction protocol and sample type. The mechanism underlying the increased Ct values observed following carrier RNA supplementation during silica membrane-based workflow of tissue samples remains unclear. One possible explanation is competition between carrier and viral RNA for silica membrane binding sites, particularly in complex tissue matrices. However, this mechanism was not directly investigated, and effects of inhibitors, matrix components, or downstream amplification cannot be excluded. Previous investigations have similarly reported that the effect of carrier RNA depends on the extraction chemistry and the characteristics of the sample [16,17].

For FMDV molecular diagnostics, neither silica membrane- nor magnetic bead-based extraction is universally superior, and method selection should consider both sample matrix and laboratory workflow requirements. Automated magnetic bead-based systems may offer operational advantages in high-throughput laboratories despite comparable amplification performance [18].

An additional practical finding of the present study was that pre-analytical storage conditions significantly influenced FMDV RNA detectability. However, it should be noted that carrier RNA served different experimental purposes in the extraction and stability experiments. In the extraction experiment, carrier RNA was included during the extraction procedure to assess its effect on extraction performance, whereas in the stability experiment, it was added directly to serum before storage and omitted during subsequent extraction. The latter experiment therefore evaluated whether the presence of carrier RNA during storage was associated with preservation of subsequent FMDV RT-qPCR detectability rather than its conventional role in facilitating nucleic acid extraction. Serum samples stored at −20 °C remained suitable for RT-qPCR analysis throughout the 60-day observation period, whereas prolonged storage at room temperature resulted in progressively increasing Ct values, particularly after 30 and 60 days. Although carrier RNA supplementation partially mitigated this loss of signal, it did not completely prevent RNA degradation.

In contrast, under the tested conditions, up to five freeze–thaw cycles did not produce a progressive loss of FMDV RT-qPCR detectability. However, the initial Ct values were approximately 17–18, representing a relatively high target concentration. A modest loss of viral RNA may therefore have little effect on detectability under these conditions but could be diagnostically relevant in weakly positive clinical specimens with viral loads approaching the assay detection limit. RNA degradation is strongly influenced by storage temperature and duration, while low temperatures generally preserve RNA integrity [19,20,21,22]. Although viral RNA stability varies depending on the biological matrix and storage medium, prolonged exposure to higher temperatures generally has a greater impact on RNA integrity than appropriate frozen storage, while the effect of repeated freeze–thaw cycles appear to be context-dependent [19,21,22,23]. Although unnecessary freeze–thaw cycles should generally be avoided as part of good sample-management practice, the present results indicate that up to five cycles did not materially impair FMDV RT-qPCR detectability under the tested conditions.

The present study has several limitations that should be considered when interpreting the findings. First, the experiments were performed using experimentally spiked matrices rather than naturally infected clinical specimens. As inactivated FMDV preparations were used, the potential influence of virus inactivation on RNA integrity, virion structure, or the extraction reagents cannot be excluded. In particular, PBS-based swab suspensions represent standardized experimental matrices and may not fully reproduce the biological complexity and potential inhibitory components of swab samples. Similarly, although bovine tissue homogenates provide a more complex biological matrix, spiking after homogenization does not completely reproduce the heterogeneous distribution of viral material or its association with cellular debris, proteins, and other tissue components present in naturally infected specimens.

Furthermore, only one commercial extraction workflow representing each extraction principle was evaluated. Because the extraction principle, commercial kit, and automated platform differed simultaneously between the two workflows, their individual contributions to the observed Ct differences cannot be separated. The findings therefore apply to the specific workflows tested and should not be generalized to silica membrane- or magnetic bead-based extraction technologies as a whole.

The experimental replicates were technical rather than independent biological replicates. Therefore, although the observed ΔCt values indicate the magnitude and direction of differences under the tested experimental conditions, statistical significance should not be interpreted as evidence of broader reproducibility or diagnostic relevance. Confirmation using independent biological replicates and naturally infected clinical specimens would be required to establish the broader diagnostic significance of these differences.

RNA yield and purity were not independently quantified; therefore, differences in Ct values reflect overall RT-qPCR performance and cannot be attributed specifically to differences in RNA recovery. The study was not designed to determine the limit of detection, probability of detection, diagnostic sensitivity, or diagnostic specificity of the RT-qPCR assay.

Finally, the storage stability and freeze–thaw experiments were restricted to a single FMDV isolate (O1 Manisa), serum matrix, and starting virus concentration. Consequently, these findings should not be directly generalized to other FMDV serotypes, strains, or sample matrices, and further studies incorporating additional viral preparations, diagnostically relevant matrices, and viral concentrations would be valuable to determine their broader applicability. Refrigerated storage at approximately 4 °C, which is relevant to short-term transport and diagnostic sample handling, was not evaluated and should be addressed in future studies.

This study highlights the critical importance of pre-analytical variables for reliable molecular detection of FMDV. Matrix-specific selection of extraction methods, careful validation of carrier RNA supplementation, appropriate frozen storage, and standardized sample handling may help maintain consistent FMDV RT-qPCR detectability. Under the experimental conditions tested, the effects of extraction workflow and carrier RNA supplementation on FMDV RT-qPCR detectability differed according to the sample matrix. These findings support matrix-specific evaluation of pre-analytical procedures rather than the assumption of a universally optimal extraction approach. Further studies using independent biological replicates, naturally infected clinical specimens, additional FMDV strains and viral concentrations, and a broader range of storage conditions are required to determine the wider diagnostic applicability of these observations.

## Figures and Tables

**Table 1:** Summary of pairwise comparisons of FMDV RT-qPCR detection according to sample matrix, extraction method, and carrier RNA supplementation.

Matrix	Comparison	Mean ΔCt	Holm-Adjusted *p*	Key Finding
Serum	Silica vs. magnetic, no carrier RNA	−6.63	0.047	Lower Ct with silica membrane-based
	Silica vs. magnetic, + carrier RNA	−3.15	0.047	Lower Ct with silica membrane-based
	Carrier vs. no carrier, magnetic	−3.51	0.094	Lower Ct with carrier; NS
	Carrier vs. no carrier, silica	−0.03	1.000	No effect
PBS-based swab suspension	Silica vs. magnetic, no carrier RNA	−1.32	1.000	No significant difference
	Silica vs. magnetic, + carrier RNA	−0.87	1.000	No significant difference
	Carrier vs. no carrier, magnetic	−0.37	1.000	No significant effect
	Carrier vs. no carrier, silica	+0.08	1.000	No significant effect
Tissue	Silica vs. magnetic, no carrier RNA	−0.89	1.000	No significant difference
	Silica vs. magnetic, + carrier RNA	+3.71	0.047	Lower Ct with magnetic bead-based
	Carrier vs. no carrier, magnetic	−2.44	0.191	Lower Ct with carrier; NS
	Carrier vs. no carrier, silica	+2.16	0.047	Carrier impaired detection

Negative ΔCt values indicate lower Ct values for the first condition relative to the reference condition; positive values indicate higher Ct values. *p*-values were adjusted for multiple comparisons using the Holm method. NS, not statistically significant.

## Data Availability

The original contributions presented in this study are included in the article/Supplementary Materials. Further inquiries can be directed to the corresponding author.

## Supplementary Materials

The following supporting information can be downloaded at https://www.mdpi.com/article/10.3390/vetsci13101096/s1, Table S1: Experimental design and raw RT-qPCR Ct data for the evaluation of FMDV RNA extraction, storage stability, and repeated freeze–thaw cycles; Table S2: Pairwise statistical comparisons of FMDV RNA extraction methods and carrier RNA supplementation across sample matrices.

## Abbreviations

The following abbreviations are used in this manuscript:

FMD	Foot-and-mouth disease
FMDV	Foot-and-mouth disease virus
RT-qPCR	Real-time reverse transcription quantitative polymerase chain reaction
RNA	Ribonucleic acid
PBS	Phosphate-buffered saline
Ct	Cycle threshold
SD	Standard deviation
IPC	Internal positive control
RT	Room temperature
